# A Case of Syphilis With Ocular Involvement: Persistent Negative Serology in a Patient With Multiple Sclerosis

**DOI:** 10.1093/ofid/ofae563

**Published:** 2024-10-29

**Authors:** Luca Pipitò, Irene Ganci, Andrea Cicero, Alice Annalisa Medaglia, Simona D’Avenia, Edoardo Mandalà, Cinzia Calà, Paola Di Carlo, Antonio Cascio

**Affiliations:** Department of Health Promotion, Mother and Child Care, Internal Medicine and Medical Specialties “G D’Alessandro,” University of Palermo, Palermo, Italy; Infectious and Tropical Disease Unit, Azienda Ospedaliera Universitaria Policlinico “P. Giaccone,” Palermo, Italy; Checkpoint Palermo Fast-Track City, Palermo, Italy; Department of Health Promotion, Mother and Child Care, Internal Medicine and Medical Specialties “G D’Alessandro,” University of Palermo, Palermo, Italy; Infectious and Tropical Disease Unit, Azienda Ospedaliera Universitaria Policlinico “P. Giaccone,” Palermo, Italy; Checkpoint Palermo Fast-Track City, Palermo, Italy; Department of Health Promotion, Mother and Child Care, Internal Medicine and Medical Specialties “G D’Alessandro,” University of Palermo, Palermo, Italy; Infectious and Tropical Disease Unit, Azienda Ospedaliera Universitaria Policlinico “P. Giaccone,” Palermo, Italy; Checkpoint Palermo Fast-Track City, Palermo, Italy; Infectious and Tropical Disease Unit, Azienda Ospedaliera Universitaria Policlinico “P. Giaccone,” Palermo, Italy; Checkpoint Palermo Fast-Track City, Palermo, Italy; Department of Health Promotion, Mother and Child Care, Internal Medicine and Medical Specialties “G D’Alessandro,” University of Palermo, Palermo, Italy; Microbiology and Virology Unit, Department of Health Promotion, Mother and Child Care, Internal Medicine and Medical Specialties “G D’Alessandro,” University of Palermo, Palermo, Italy; Microbiology and Virology Unit, Department of Health Promotion, Mother and Child Care, Internal Medicine and Medical Specialties “G D’Alessandro,” University of Palermo, Palermo, Italy; Department of Health Promotion, Mother and Child Care, Internal Medicine and Medical Specialties “G D’Alessandro,” University of Palermo, Palermo, Italy; Infectious and Tropical Disease Unit, Azienda Ospedaliera Universitaria Policlinico “P. Giaccone,” Palermo, Italy; Checkpoint Palermo Fast-Track City, Palermo, Italy; Department of Health Promotion, Mother and Child Care, Internal Medicine and Medical Specialties “G D’Alessandro,” University of Palermo, Palermo, Italy; Infectious and Tropical Disease Unit, Azienda Ospedaliera Universitaria Policlinico “P. Giaccone,” Palermo, Italy; Checkpoint Palermo Fast-Track City, Palermo, Italy

**Keywords:** multiple sclerosis, negative syphilis serology, ocular syphilis, ofatumumab, otosyphilis

## Abstract

Diagnostic algorithms for syphilis are based on serology. However, with the advent of immunosuppressive therapies, these algorithms may fail. We report a case of an individual with multiple sclerosis on treatment with ofatumumab and secondary syphilis with visual and auditory systems involvement and persistent negative treponemal and nontreponemal tests.

Syphilis is a reemergent disease, and its incidence has increased in recent decades [1, 2]. The clinical picture can be varied and misleading, and every type of skin manifestation can be associated with syphilis during the secondary stage [2]. The central nervous system and ocular and vestibulocochlear organs can be involved at any stage of syphilis [2]. Diagnosing syphilis can be challenging, as the disease is known as “the great imitator” due to its ability to mimic the symptoms of various other conditions. Traditional and reverse sequence diagnostic algorithms are based on serology that is distinguished between nonspecific (nontreponemal tests, such as rapid plasma reagin [RPR] or Venereal Disease Research Laboratory test [VDRL]) and specific (treponemal test) [3]. These algorithms have limitations, particularly in recent infections where serology may be negative [3], and they can fail in immunocompromised individuals [4, 5]. We present a case of a patient with multiple sclerosis on monoclonal antibody therapy who developed secondary syphilis with suspected visual and auditory systems involvement. In this patient, syphilis serology remained negative, and only molecular biology confirmed the diagnosis after a prolonged clinical course lasting several months.

## CASE REPORT

### Medical History

In March 2024, a 37-year-old man presented to the infectious disease clinic with a 3-month history of several symptoms. His medical history was remarkable for multiple sclerosis in treatment with monthly ofatumumab from July 2023, seborrheic dermatitis, psoriasis, and hemorrhoid disease. He was a general practitioner and reported a detailed anamnesis about his condition.

### History of Clinical Manifestations

On 20 January 2024, he reported a self-resolved itching penile erythema with a burning sensation, and after a few days, he complained of bloody diarrhea and anorectal pain. In the next few days, he developed an itching desquamative rash involving the right eyebrow with hair loss. The rash diffused to the face, involving the beard and hair. He noticed the appearance of an anal painless nodular lesion, which he treated as a hemorrhoidal thrombosis with topical medicaments without improvement. Within 2 weeks, the rash scattered to the arms, limbs, and trunks. The skin manifestations were varied. He observed macules, papules, and erythematous plaques with raised scaly borders, some ulcerated, and oval patches with fine scale along the borders. The rash initially was nonpruritic and involved palms and soles. Thereafter, the rash became itchy, and he was referred to a dermatologist who suspected guttate psoriasis. Topical therapy was not effective. On 7 March, he learned that his ex-partner (male) had tested positive for syphilis. Consequently, he underwent testing and began treatment with doxycycline. After 2 days, he developed a high fever, chills for 2 days, and myalgias. The treponemal test was negative, and due to the persistence of the lesions, he presented to the infectious diseases clinic. He avoided taking the expected dose of ofatumumab scheduled for March.

### Presentation at Our Clinic

Physical examination was remarkable for the skin rash described above. Serology for human immunodeficiency virus (HIV) (antigen and antibodies), hepatitis C virus (antibodies), hepatitis B virus (antibodies to hepatitis B surface antigen, hepatitis B surface antigen, anti-core), syphilis (treponemal and RPR), and multiplex polymerase chain reaction (PCR) assay (chlamydial, gonococcal, *Mycoplasma*, *Ureaplasma*, and *Trichomonas* infections) on urethral and rectal swabs were performed. A chemiluminescent immunoassay (DiaSorin) was used to detect treponemal antibodies (immunoglobulin G and immunoglobulin M [IgM]) and the BD Macro-Vue RPR card test for RPR. The tests had negative results, but the patient continued to take doxycycline for 25 days.

In the following days, the patient developed conjunctival hyperemia and photophobia with a subsequent decrease of vision in the right eye, and after that he complained of the onset of intermittent tinnitus, which he had never experienced before. On 6 April, he had an ophthalmological consultation, which led to a presumptive diagnosis of viral conjunctivitis and noted the presence of inferior vitreous vascular veils.

### Admission to the Hospital

**Figure 1.**
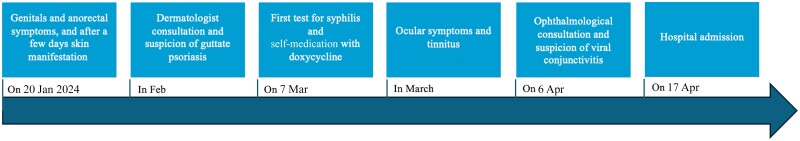
Timeline of the patient's history, summarizing the onset of clinical manifestations and medical consultations.

**Figure 2.**
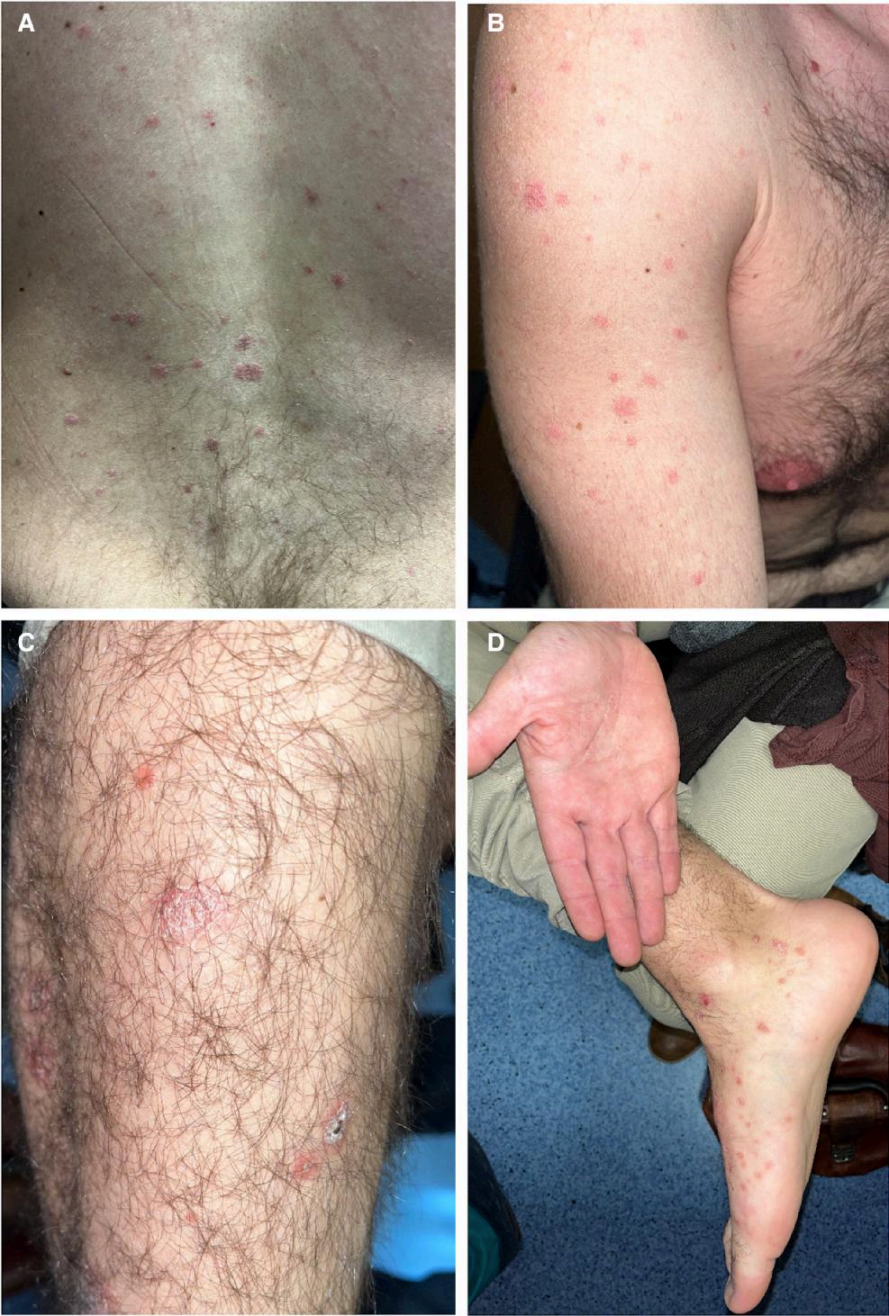
Physical examination on admission revealed scattered red maculopapular lesions, some with scaly edges and some crusted. Lesions are seen on the back (*A*), arms (*B*), lower limbs (*C*), and feet and palms (*D*).

**Figure 3.**
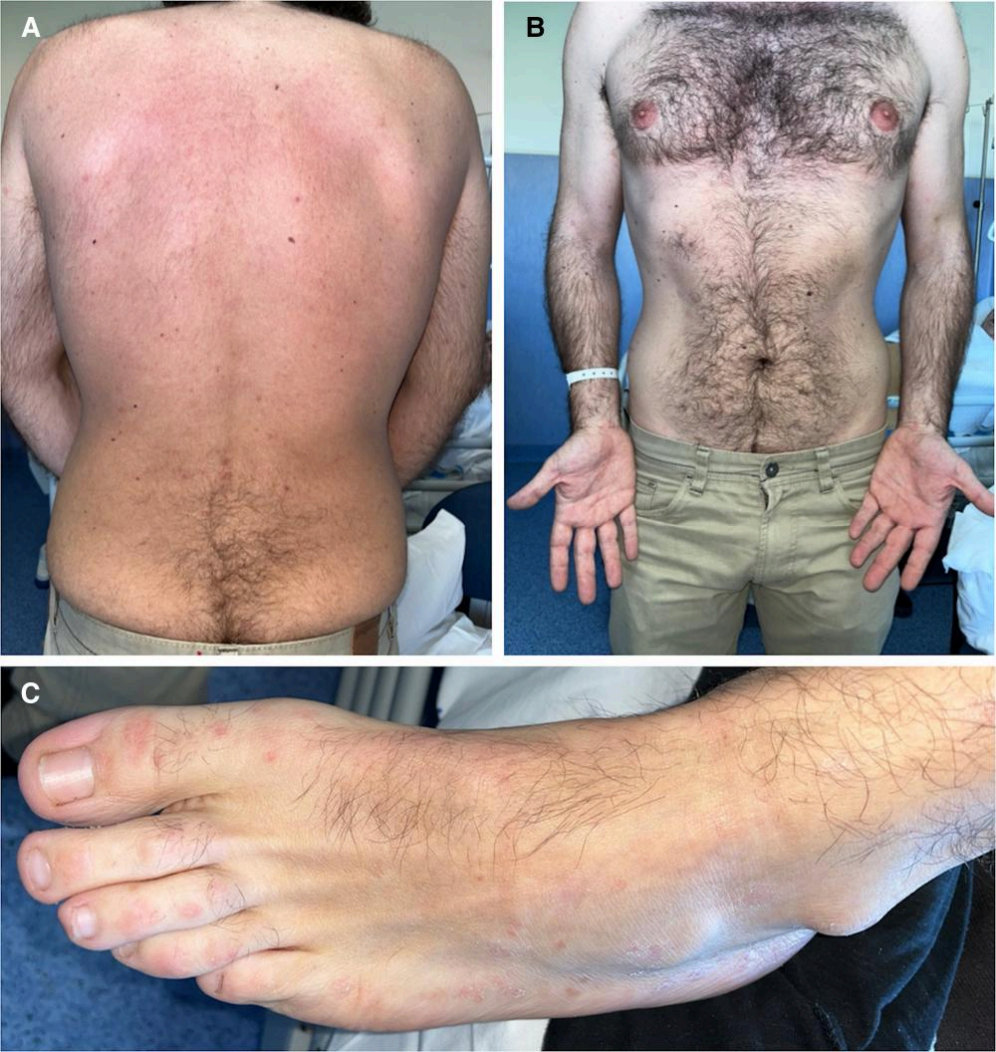
Physical examination on discharge showed resolution on the skin lesions on the back (*A*) and trunk and arms (*B*). Some pigmented red lesions lighter than the original ones persisted, especially on the feet (*C*).

On 17 April, due to the persistence of symptoms and the suspicion of ocular syphilis and otosyphilis, the patient was admitted to the hospital. A timeline of the patient's history is depicted in Figure 1. Physical examination showed nummular erythematous lesions scattered in all the body (Figure 2), including palms and feet. Some lesions were flat, while others were slightly raised (small plaques and red scaling papules). Certain lesions exhibited peripheral scaly collarettes, while others were crusted. No penile lesions and lymphadenopathies were observed, and a small (<1 cm), painless, and buttonlike consistency on palpation nodular anal lesion was highlighted. The patient’s body temperature was 36.5°C, blood pressure was 144/85 mm Hg, pulse rate was 85 beats per minute, oxygen saturation was 99% on ambient air, and respiratory rate was 16 breaths per minute. Chemical examination was unremarkable except for mild hypogammaglobulinemia of 6 g/dL (reference range, 6.7–15.7 g/dL). The white blood cell count was 6.14 × 10^3^ cells/μL, with 63.1% neutrophils (reference range, 40%–74%), 23.3% lymphocytes (reference range, 20%–48%), and 9.1% monocytes (reference range, 3%–11%). Treponemal and RPR serology showed a further negative result. The patient declined a lumbar puncture to evaluate for neurosyphilis. Treatment with 24 million units/day of intravenous crystalline penicillin G was started. The magnetic resonance imaging performed during hospitalization was comparable to the previous ones, showing a stable picture of multiple sclerosis. A multiplex real-time PCR (Genital Ulcer Assay [Allplex]) identifying herpes simplex virus type 1/2, *Haemophilus ducreyi*, cytomegalovirus, *Chlamydia trachomatis* serovars L1–L3, *Treponema pallidum*, and varicella zoster virus was performed on the rectal swab collected previously during the first outpatient visit and turned positive for *Treponema pallidum*, confirming the diagnosis of syphilis. After 14 days of intravenous penicillin G, a single dose of 2.4 million units of intramuscular benzathine penicillin G was administered. Both treponemal and RPR serology remained negative at discharge. However, skin lesions improved significantly (Figure 3) and ocular and auditory symptoms regressed. Ten days after discharge, the patient developed *Clostridioides difficile* colitis, which was successfully treated with oral vancomycin. Two months later, the patient did not have any skin lesions during the follow-up visit. Ofatumumab administration was resumed on 29 June. He repeated the treponemal test and RPR serology, which both continued to be negative.

## DISCUSSION

We reported a case of secondary syphilis with ocular and auditory system involvement of a patient with multiple sclerosis on treatment with ofatumumab and persistent negative syphilis serology.

### The Great Imitator

Syphilis has historically been termed the “great imitator” or “the great impostor” due to its remarkable ability to mimic a wide range of clinical manifestations and involve nearly any organ system [2]. The primary lesion, known as a chancre, typically appears at the site of *Treponema pallidum* penetration. A chancre usually presents as a painless ulcer that might go unnoticed, but its appearance can vary; it can be painful or even present as multiple lesions [2, 6]. In our case, the patient developed a nodular anal lesion, which he mistakenly thought was hemorrhoid disease. Secondary syphilis is usually marked by diffuse skin manifestations, indicative of the dissemination of *Treponema pallidum*. Skin lesions can take on various forms and are easily confused with other types of rashes or skin conditions [2, 6]. These characteristics lead clinicians to make diagnostic errors, and our case is an example of this. Initially, a dermatologist, likely misled by the patient's medical history of psoriasis vulgaris, suspected that the clinical manifestations might be related to a case of guttate psoriasis. Later, an ophthalmologist considered that the onset of ocular symptoms could be due to a viral infection. Our case was complicated by the patient's medical history, which included multiple sclerosis, psoriasis, and treatment with a monoclonal antibody; however, clinical signs, such as loss of eyebrow hair (“omnibus sign”), the presence of diffuse skin lesions with varying appearances, and involvement of the palms and soles, should always raise suspicion of syphilis in any patient [7].

### Syphilis With Seronegative Serology

Ofatumumab is a fully human monoclonal antibody that targets CD20, a protein expressed on the surface of B lymphocytes used for the treatments of chronic lymphocytic leukemia and relapsing forms of multiple sclerosis [8]. It works by binding to the CD20 protein on B cells, leading to their depletion, and its use was associated with a reduction of the mean serum IgM level [8]. In our case, the patient continued ofatumumab administration until the scheduled dose of March, and it probably influenced the persistent negative result for syphilis. Atypical clinical and serological courses of syphilis were observed in immunocompromised individuals, especially in people with HIV with false-negative serology [4], but no data were reported about the effect of ofatumumab. A previous case of syphilis with initially negative serology was reported in a patient with multiple sclerosis on treatment with rituximab, which is another anti-CD20 [5]. Unlike our case, the treponemal test showed a delayed and weak seroconversion (2.35, positive >1, ARCHITECT Syphilis TP assay), while RPR persisted nonreactive.

### The Need for New Diagnostic Algorithms for Syphilis

With the advent of monoclonal antibodies with immunosuppressive action for the treatment of hematological malignancies or autoimmune diseases, new diagnostic algorithms for syphilis are necessary. In these cases, molecular biology plays an essential role and can be performed on mucosal swabs, as in our case, or on skin biopsies, as described in only 1 previous report [5]. A rash compatible with secondary syphilis should not be underestimated, regardless of serology, in individuals receiving monoclonal antibody therapy, as documented in our patient. Another crucial point for this class of patients is the follow-up. When faced with negative nontreponemal serology, it is not feasible to conduct follow-up based on the reduction of RPR/VDRL titers to demonstrate recovery. In cases like ours, only clinical monitoring is currently feasible. However, this approach does not allow us to exclude the possibility of reservoirs in the central nervous system or latent forms that could potentially progress to the tertiary stage over time.

### Treatment Considerations

Another interesting aspect of our case is the lack of response to doxycycline treatment, which the patient took before the appearance of ocular symptoms. Penicillin is recommended as the first-line treatment for syphilis according to both European and American guidelines, whereas doxycycline is recommended as an alternative for patients with penicillin allergy [3, 9]. Additionally, most patients with penicillin allergies can be safely desensitized [10]. Thus, penicillin should be considered the preferred option whenever feasible. The patient likely chose doxycycline due to its ease of self-administration compared to intramuscular penicillin injections. It is unclear whether this nonresponsiveness was due to *Treponema pallidum* resistance or the presence of a neurological reservoir. Only 1 previous report identifying resistance to tetracycline by mutation analysis was reported in a metanalysis [11]. However, doxycycline is a bacteriostatic antibiotic, and in cases like ours, where there is an immunodeficiency, it may not be sufficient to eradicate the infection. Therefore, it seems prudent to use penicillin in such cases rather than a second-line drug.

## CONCLUSIONS

In conclusion, to the best of our knowledge, this is the first reported case of nonreactive syphilis in an HIV-negative individual undergoing treatment with ofatumumab. We believe that such cases are likely to become more common in the future. Therefore, clinicians must exercise caution when encountering clinical presentations suggestive of syphilis in patients on treatment with anti-CD20, regardless of the features of skin lesions, which can vary during secondary syphilis. In such cases, molecular biology techniques are crucial for confirming the suspected diagnosis.

## Notes

***Author contributions.*** L. P.: Writing and conceptualization. I. G., A. Ci., S. D., A. A. M., and P. D. C.: Visualization. E. M. and C. C.: Microbiological examination. A. Ca: conceptualization and revision.

***Patient consent.*** Informed consent was obtained from the patient for the publication of all images, clinical data, and other data included in the manuscript.
